# E-cigarette Aerosol Containing Nicotine Increases Aortic Stiffness in Young Mice

**DOI:** 10.1007/s12012-026-10156-1

**Published:** 2026-07-08

**Authors:** Pireyatharsheny Mulorz, Joscha Mulorz, Agnesa Mazrekaj, Hsiang-Han Liu, Wiebke Ibing, Joshua M. Spin, Lasse Bach Steffensen, Hans C. Beck, Uwe Raaz, Kensuke Toyama, Hubert Schelzig, Philip S. Tsao, Markus U. Wagenhäuser

**Affiliations:** 1 Division of Cardiovascular Medicine, Stanford University, Stanford, CA USA; 2 VA Palo Alto Health Care System, Palo Alto, CA USA; 3 Clinic for Vascular and Endovascular Surgery, University Hospital Düsseldorf and Medical Faculty, Heinrich-Heine-University, Düsseldorf, Germany; 4 Department of Molecular Medicine, University of Southern Denmark, Odense, Denmark; 5 Department of Clinical Biochemistry, Odense University Hospital, Odense, Denmark; 6 Department of Cardiology and Pneumology, University Medical Center Göttingen, Georg-August-University, Göttingen, Germany; 7 German Center for Cardiovascular Research (DZHK), Partner Site, Göttingen, Germany; 8 University Heart Center, Göttingen, Germany; 9 Division of Cardiovascular Surgery, Department of Surgery, Kurume University School of Medicine, Kurume, Japan; 10 Cardiovascular Center, Kurume University Hospital, Kurume, Japan

**Keywords:** Aortic stiffness, E-cigarette, Vascular remodeling, Electronic cigarette, Vaping, Nicotine

## Abstract

E-cigarettes (E-cigs) are widely used especially among young people, but the effects on vascular stiffening and remodeling are poorly understood. This study evaluates the effects of short-term e-cig exposure on young vs. adult mice and the effects of e-cig derived chemicals present in plasma and urine of exposed animals on cultured endothelial cells (EC) and vascular smooth muscle cells (SMC). C57BL/6J mice of 6- and 14-weeks of age were exposed to unflavored e-cig aerosol with and without nicotine (50% propylene glycol, 50% vegetable glycerol +/− 24 mg/ml nicotine 3 weeks, puff duration of 9 s per minute, for 60 min/day) and aortic tissue subjected to in- and ex-vivo assessment of stiffness and histological evaluation. Major chemical constituents of e-cig aerosol were analyzed in plasma and urine samples, and cultured human aortic ECs and SMCs were treated with these chemicals, including acrolein, formaldehyde and nicotine. Changes in gene and protein expression were quantified, and functional ROS and MMP assays were performed. Pulse wave velocity revealed increased stiffness upon treatment with e-cig aerosol, and to a greater extent with the inclusion of nicotine (114% in 6-week-old and 112% in 14-week-old mice vs. baseline, *p* < 0.001), which was also observed in ex-vivo myography in aortic infrarenal and thoracic segments in 6-week-old mice after e-cig exposure. Aortic elastin content in these mice was decreased when compared to room-air controls (-24% in 6-week-old and − 36% in 14-week-old mice in thoracic aorta, *p* < 0.05). In vitro treatment with some of the different chemical compounds present in e-cig aerosol led to an increase in endothelial activation markers, and extracellular remodeling proteins. Wire myography showed an endothelium-independent decrease in relaxation in young murine aortas treated with nicotine. Our results indicate that short-term exposure over three weeks to e-cig aerosol leads to marked changes in murine aortic stiffness and vascular remodeling, potentially predisposing for cardiovascular disease conditions despite the superior remodeling capabilities of young animals.

**Graphical Abstract:**

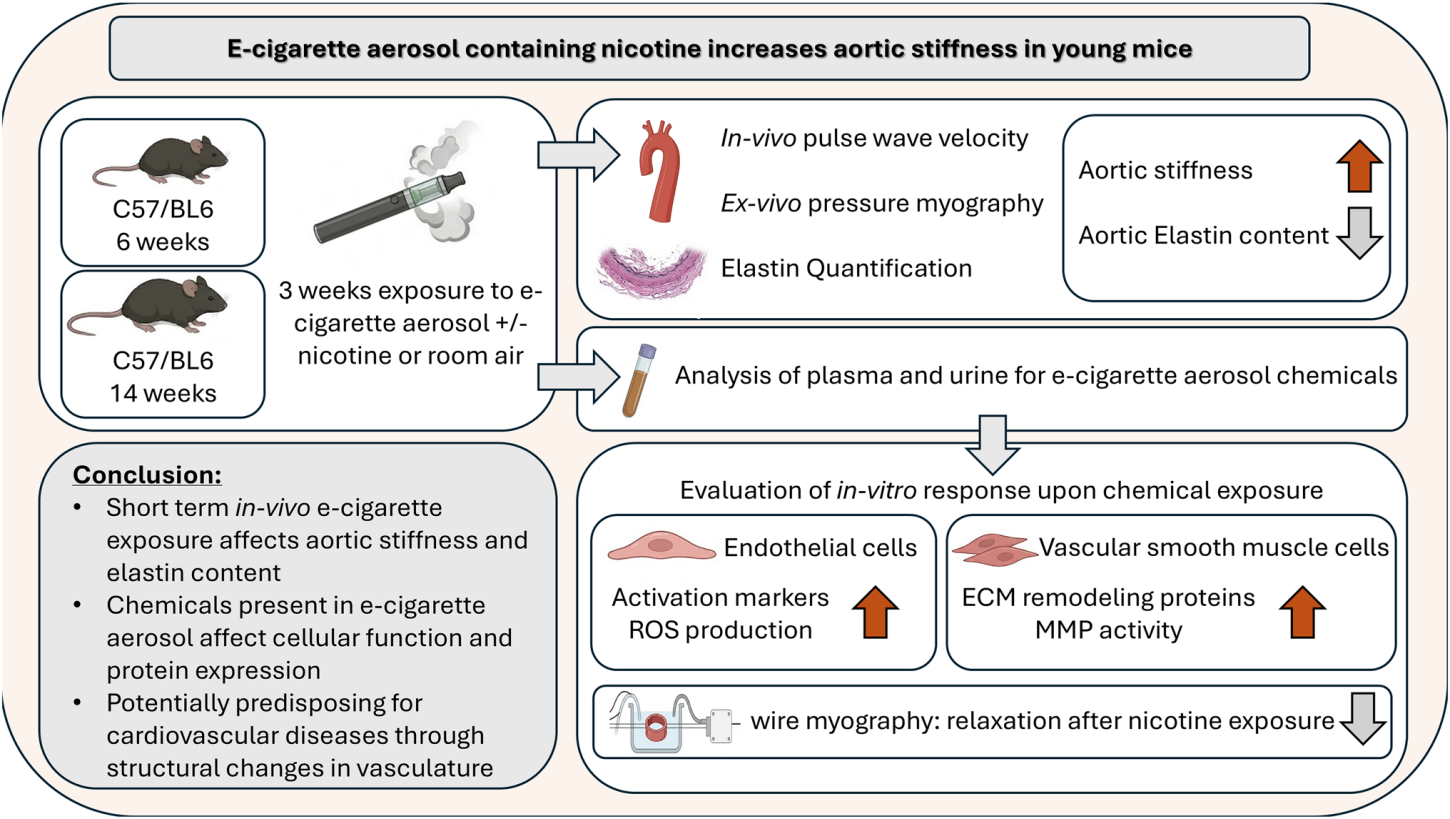

**Supplementary Information:**

The online version contains supplementary material available at 10.1007/s12012-026-10156-1.

## Introduction

Modifiable behavioral risk factors are leading causes of mortality in the United States. Among them, cigarette smoking has been identified as the leading preventable cause of death, killing more people than HIV, illegal drug and alcohol consumption, motor vehicle accidents and firearms combined [1, 2]. Though the rates of conventional cigarette smokers have been declining in recent years, the number of e-cigarette (e-cig) users has rapidly increased [3]. Initially introduced in 2003, e-cigs have gained popularity with all ages, sexes and ethnicities [4, 5]. This is especially true for young people. E-cigs have become more commonly used among 12th graders than tobacco cigarettes [6]. Young people are the group with the highest increase in usage, with 5.3% of all users being middle-schoolers and 16% being high school students, resulting in 9- and 10-fold increases, respectively, since 2011 [6]. According to a 2016 report by the U.S. Surgeon General, 13.5% of middle school students, 37.7% of high school students, and 35.8% of young adults (18 to 24 years of age) have used e-cigs, compared with 16.4% of older adults (25 years and up) [7].

E-cigs work by heating a solution (known commonly as “e-liquid” or “e-juice”) usually containing nicotine as its active ingredient, as well as propylene glycol and glycerol [8]. However, e-cigs are largely unregulated and are manufactured by various companies. Therefore, the contents of e-cigs vary widely and in some cases are not consistent with labeling [9, 10]. While there is a general public conception that e-cigs are relatively harmless, as they lack toxic tobacco combustion products, the nicotine content in e-cigs can exceed the delivery profile of a tobacco cigarette [11, 12]. Little is known about the health effects of e-cigs. Perceptions of potential risks and benefits of e-cig use vary widely among the public, users of the products, health care providers, and the public health community [13].

In the realm of cardiovascular medicine, recent human studies have found that e-cigs can increase aortic stiffness [14]. Aortic stiffness has been identified as a strong independent risk factor for many cardiovascular conditions, including heart failure, myocardial infarction, stroke, and abdominal aortic aneurysm [15]. Stiff conduit arteries lose their capability to mechanically buffer against the pulsatile nature of cardiac ejections, resulting in widespread augmentation of hemodynamic stress on end-organs. Notably, increased aortic stiffness has also been observed in young e-cig users, increasing concern for exposure in younger generations [16].

While e-cigs can induce short-term effects leading to temporary stiffness changes, mostly due to the inhibition of vasodilation, there are also long-term effects due to changes in the vessel architecture. So far there are few published studies examining the influence of e-cig vapor on cells of the vascular tree. There is some evidence that exposure can lead to increased stress-response and cell death in endothelial cells [17, 18]. Our group and others have shown enhanced aortic inflammation and increased stiffening due to mid- and long-term exposure to e-cig aerosol in various in-vivo exposure experimental settings [19–21]. E-cig exposure has also been shown to induce enhanced systemic inflammation in murine models, and cardiac inflammation in a rat model of myocardial infarction [22, 23].

There is great concern that exposure to potential toxins during adolescence may result in even greater harm than exposure in adulthood, given the vulnerability of this population to the acute and chronic effects of toxins in general, and from their cumulative exposure if started at young age [24]. Recent studies in this context showed that young regular users of e-cigarettes exhibit reduced vascular function primarily in the microvasculature [25].

Despite the aforementioned evidence linking e-cig exposure to increased vascular stiffness and oxidative stress in preclinical models and few clinical studies, no published work has yet investigated these effects in young animals as opposed to adult or aged cohorts.

Given that e-cig devices are often advertised as healthier alternatives to conventional cigarettes [26] and their popularity among young people, it would seem crucial to explore the vascular consequences of e-cig consumption at an early stage.

## Results

### E-cig Exposure to Nicotine Increases Aortic Stiffness in Mice

**Fig. 1:**
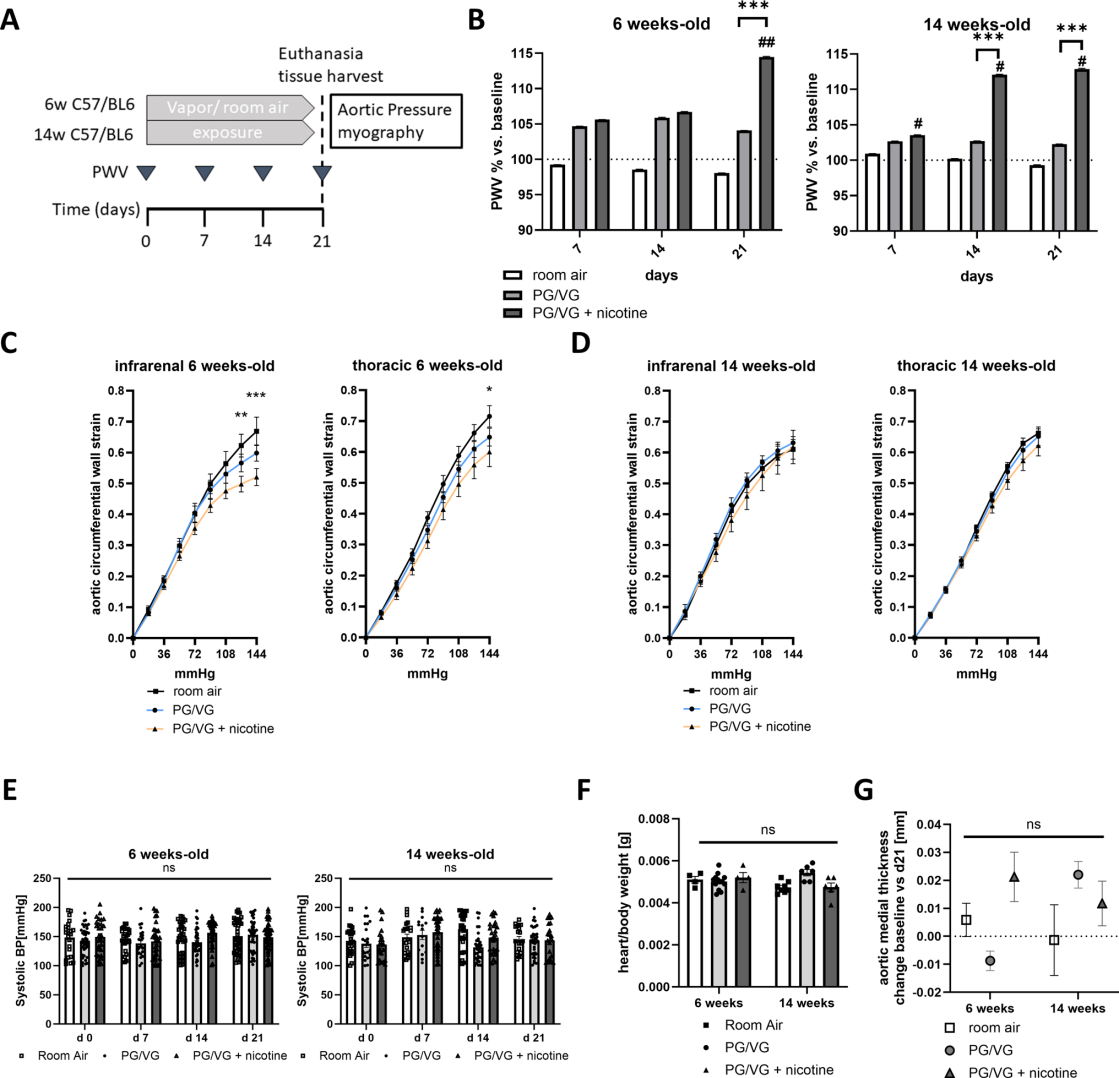
Exposure to e-cig aerosol increases aortic stiffness in mice. Overview of in-vivo study design. Young (6-week-old) and adult (14-week-old) mice were exposed to either room air or e-cig aerosol containing either PG/VG 50/50 or PG/VG 50/50 + nicotine for 3 weeks for 1 h per daily (**A**). Mice were assessed for pulse wave velocity (PWV) once per week using ultrasound (*n* = 8 per group) Data show percent increase vs. baseline (**B**). At the end of exposure, mice were euthanized and aortic stiffness in the thoracic and infrarenal aortic segment was assessed ex-vivo using pressure myography in 6-week-old mice (**C**) and 14-week-old mice (**D**). *n* = 8–10 per group. Mice were assessed for changes in blood pressure (MAP=mean arterial pressure) in response to inhaled treatments using the tail-cuff method (**E**), *n* = 4 per group. At the end of the experiment, body and heart weight were measured to calculate a ratio to examine potential changes due to elevated blood pressure (**F**), *n* = 4–13. Using M-Mode ultrasound images, aortic medial thickness was measured in the infrarenal segments and changes relative to baseline quantified (**G**), *n* = 4–8 per group. Graphs show mean with SEM or individual data points and SEM (**E**,** F**) #= *p* < 0.05 vs. room air. ##= *p* < 0.01 vs. room air and ***=*p* < 0.001 vs. PG/VG 50/50, Mann-Whitney-U test (**B**). *=*p* < 0.05, **=*p* < 0.01, ***=*p* < 0.01 PG/VG+nicotine vs. room air ANOVA with multiple comparisons (**C**). ns = *p*>0.05, t-test (**E**–**G**)

Male C57BL/6J mice of 6 and 14 weeks of age (young and adult) were exposed to e-cig aerosol with and without nicotine on a daily basis for 3 weeks, for one hour per day. During this time mice were assessed for pulse wave velocity (PWV) by ultrasound and upon sacrifice, infrarenal and thoracic aortic segments were subjected to arterial pressure myography (Fig. 1A). PWV measurements revealed a significant increase (vs. baseline) in all mice exposed to e-cig aerosol containing nicotine after 21 days of exposure. In adult mice, the increase reached significance by 7 days if the aerosol contained nicotine. Of note, when mice were exposed to PG/VG without nicotine, there was no significant increase vs. room air exposure, which indicates the importance of nicotine as a main substituent of the aerosol in this context (Fig. 1B).

When assessed with pressure myography, aortic segments from young mice exhibited less elasticity after exposure to aerosol containing nicotine, for both the infrarenal and thoracic aorta. Similar findings were observed in response to exposure to non-nicotine-containing aerosol for the infrarenal segment only, albeit not as pronounced when compared to added nicotine (Fig. 1C). No significant differences were observed in adult mice (Fig. 1D). Of note, e-cig exposure did not lead to differences in mean blood pressure when assessed with tail cuff measurements (Fig. 1E). In addition, no differences in heart-body weight ratios were observed (Fig. 1F) and aortic medial thickness measured in the ultrasound pictures for PWV measurements showed no significant change in young and adult mice in response to e-cig exposure with and without nicotine, although there was a trend towards increased thickness with nicotine vape (vs. room air) in both age groups (Fig. 1G).

### E-cig Exposure Reduces Aortic Elastin Content in Young and Adult Mice

**Fig. 2:**
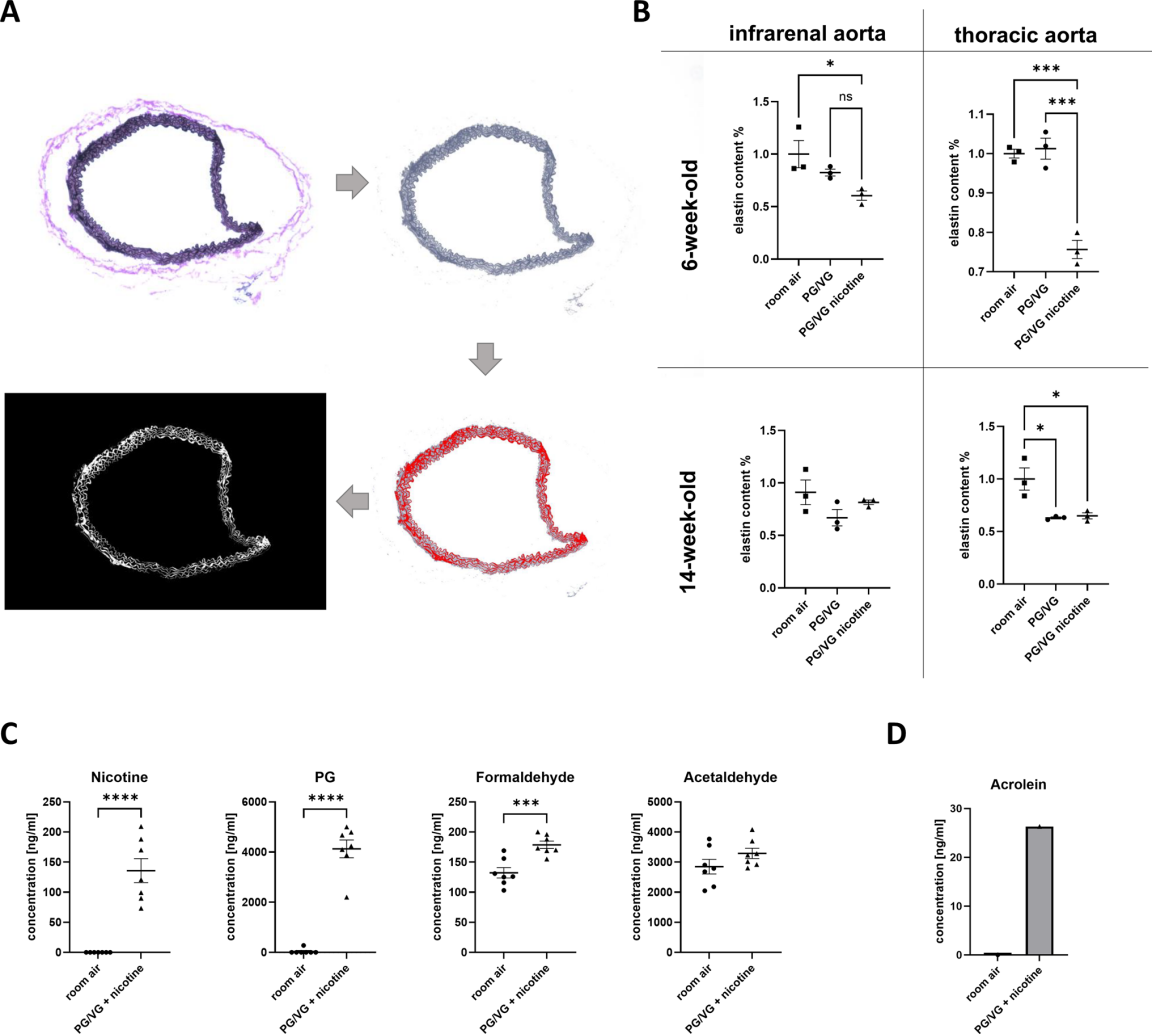
Elastin content is reduced in response to e-cigarette exposure in murine aorta and aerosol constituents in murine plasma and urine. Cross sections of murine aortas were stained with Elastica van Giesson and imaging was assessed for elastin content using an automated ImageJ-based analysis algorithm (**A**). Exposure to e-cig aerosol reduced elastin content in the thoracic segments of 14-week-old mice (upper panel) and infrarenal and thoracic segments of 6-week-old mice (lower panel) (**B**). Murine plasma was collected from 14-week-old male mice shortly after exposure to e-cig aerosol containing nicotine and concentration of major chemical components in e-cig aerosol were measured using Mass Spectrometry (**C**), *n* = 6 per group. Acrolein was measured using Mass Spectrometry in pooled urine from 6 aerosol-exposed mice vs. room air exposed mice (**D**). *n* = 3 mice per group and 2 slices analyzed per animal. *=*p* < 0.05 and ***=*p* < 0.001 vs. room air or PG/VG group, ANOVA with multiple comparisons (**A**, **B**). ***=*p* < 0.01 vs. room air group ****=*p* < 0.001 vs. room air group in t-test (**C**,** D**)

A subset of mice was exposed using the same protocol as depicted in Fig. 1A and aortic tissue was harvested from both the descending thoracic and infrarenal segments. Cross-sections were stained for elastic fibers and a semi-automated image analysis was applied to quantify content of elastin fibers per image (Fig. 2A). Elastin content was significantly reduced in the thoracic segments of adult mice exposed to e-cig vapor (vs. room air), with and without nicotine. In the young mice this effect was observed in both infrarenal and thoracic aortic segments, with significantly greater reductions in elastin content in response to e-cig exposure containing nicotine (vs. room air, or PG/VG alone) (Fig. 2B).

### Constituents of E-cig Aerosol are Increased in Murine Plasma and Urine, and Affect Human Vascular Cells

**Fig. 3:**
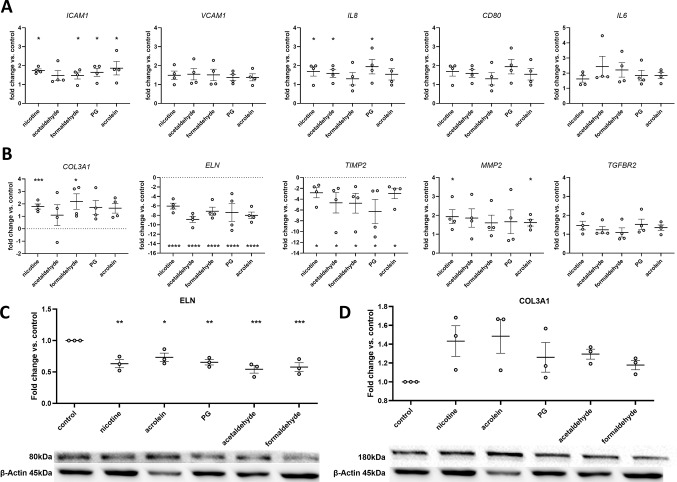
Effects of e-cig aerosol chemical constituents on vascular cells in-vitro. The effects of different chemicals analyzed in murine plasma and urine in response to e-cig exposure on gene expression of several target genes in cells of the aorta were examined in human aortic endothelial cell (**A**) and human aortic SMC’s (**B**) by qPCR, *n* = 4 per group. Results in SMC’s were confirmed using Western Blot for ELN and Col3A1, *n* = 3 per group (**C**,** D**). Data presented as mean with SEM, gene expression data are presented as fold change (FC) vs. untreated control with own-regulated genes shown as − 1/FC. *=*p* < 0.05 vs. untreated control and ****=*p* < 0.001 vs. untreated control in t-test (**A–D**). Full uncropped gels available in the Suppl. Fig. 2 for E and F

To evaluate to what extent e-cig aerosol constituents contribute to the effects observed in the mice, plasma samples from adult mice exposed to the aforementioned protocol were harvested within 30 min after their last exposure session and analyzed for major chemical constituents known to be present in vaporized e-juice. We found that plasma concentrations of nicotine, propylene glycol (PG) and formaldehyde were significantly increased when compared to room-air exposed mice (Fig. 3A). While plasma acetaldehyde levels did not differ significantly, acrolein concentrations were highly increased in pooled urine samples between treatment groups (Fig. 3B). Pooling was necessary in this case because of the small amounts of urine per animal.

We investigated the effects of the respective chemicals compounds on cells of the aortic wall, specifically human aortic endothelial cells (haoEC) and human aortic vascular smooth muscle cells (haoVSMC). Due to a lack of comparable data available from literature, it was first necessary to establish a treatment concentration for these chemicals which was likely not to harm the cells while still allowing for treatment effect detection. A commercially available assay for metabolic cell activity was used to evaluate cell-viability for both cell types to determine the highest possible concentration of each chemical for which cell viability remained over 90% (Suppl. Fig. 1 A, B).

After 24 h of treatment, haoECs were assessed for gene regulation of known pro-inflammatory surface proteins and cytokines. Here, *ICAM1* was shown to be significantly upregulated in response to almost all the chemicals. *IL8* was also significantly upregulated in response to treatment with nicotine, acetaldehyde and PG. *VCAM1*, *IL6* and *CD80* displayed consistent upregulation with all treatments, but did not reach significance (Fig. 3C). In haoVSMCs, gene regulation of proteins crucial to ECM remodeling were assessed. Here, nicotine and formaldehyde treatment significantly increased *COL3A1* expression. *MMP2* expression was significantly upregulated with nicotine and acrolein treatment. Of note, all chemicals tested strongly downregulated *ELN* with up to ~ 8-fold downregulation. *TIMP2* expression was also significantly reduced in response to chemical treatment, while *TGFBR2* expression was not altered (Fig. 3D). Results for elastin were confirmed using western blot, showing a significant decrease in ELN in response to all the chemicals tested in haoVSMCs Western blots also showed a trend in COL3A1 upregulation with chemical treatment, yet not reaching significance (Fig. 3E).

### Aerosol-Constituents Increase Cellular ROS and MMP Activity

**Fig. 4:**
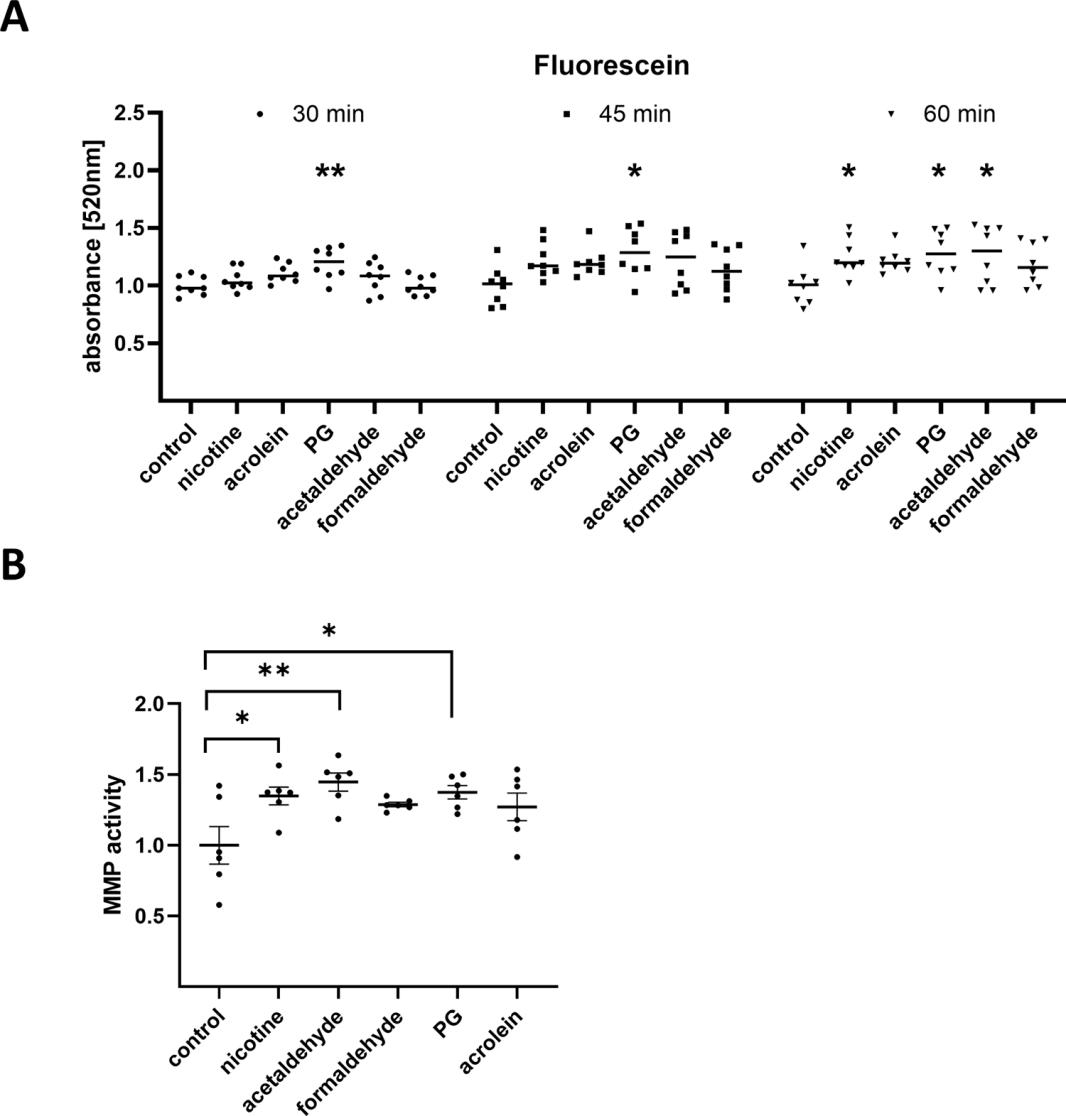
E-cig constituents affect cellular ROS production and MMP activity. Following 24-hour exposure to several e-cig-aerosol constituting chemicals, human aortic ECs were assessed for ROS production by fluoresce absorbance measurements (**A**). MMP activity was quantified after treatment with e-cig-vapor constituting chemicals in human aortic smooth muscle cells (**B**). Data show mean with SEM. *=*p* < 0.05; **=*p* < 0.01 vs. untreated control assessed by ANOVA with multiple comparison (**A**,** B**)

E-cig consumption has been repeatedly reported to induce oxidative stress, particularly in the endothelium [27, 28]. We therefore examined ROS production in haoECs following treatment with the chemicals mentioned above. Cell exposure to PG significantly increased ROS when compared to control after 30 min; in addition, nicotine and acetaldehyde also significantly increased ROS production after 60 min (Fig. 4A), while superoxide production (rhodamine) was not changed (Suppl. Fig. 1C).

Given that we observed reduced elastin content in response to e-cig vapor exposure and downregulation of *TIMP2* in response to treatment with e-cig vapor constituents, we also assessed MMP activity in haoVSMCs using a fluorometry-based commercially available kit. We observed increased MMP activity for all chemicals tested, although the increase was only statistically significant for nicotine, acetaldehyde and PG (Fig. 4B).

### Nicotine Treatment Affects Endothelium-Independent Relaxation Response Differently in Young vs. Adult Mice

**Fig. 5:**
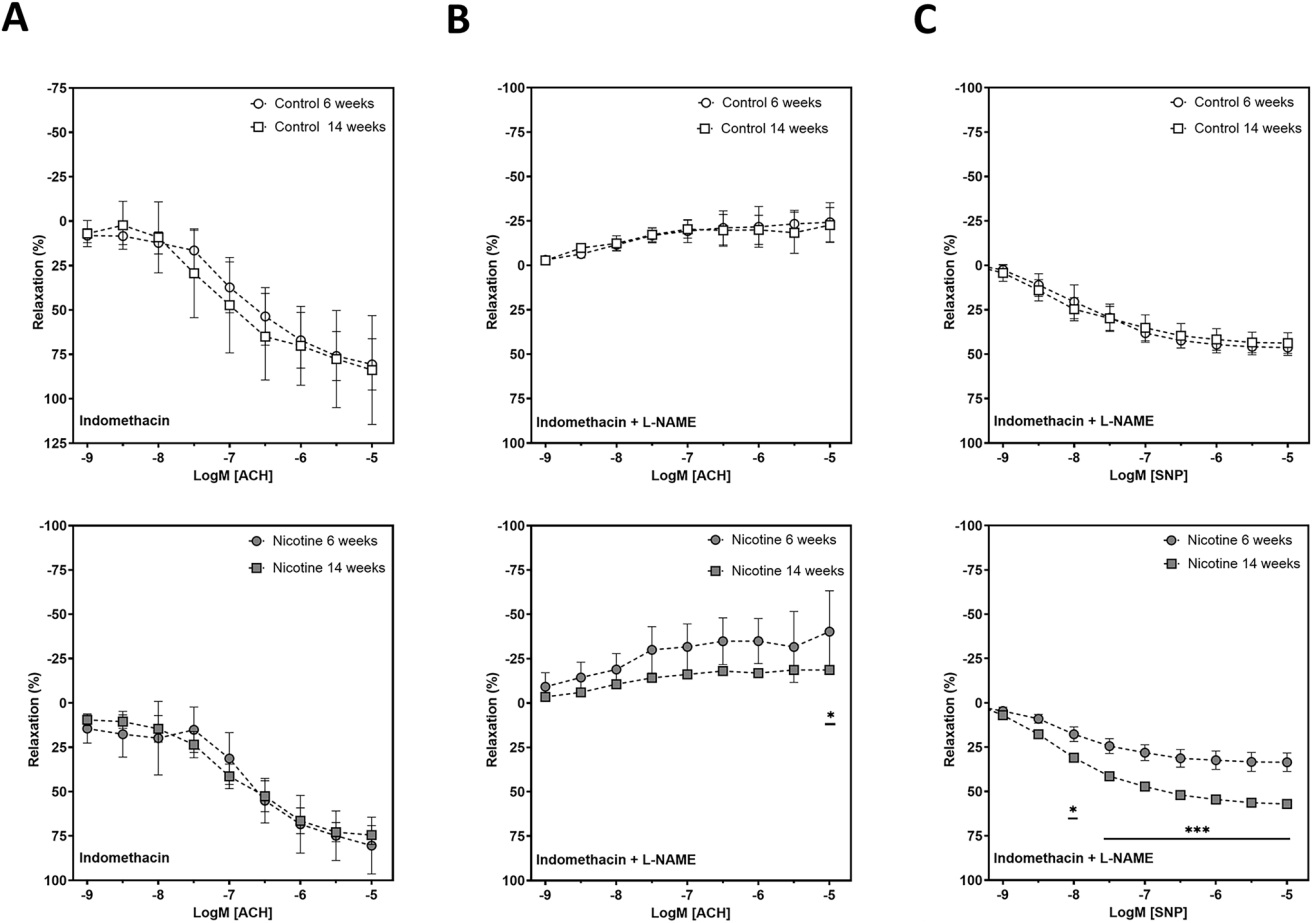
Assessment of vascular dysfunction response to nicotine in young and adult mice. Thoracic aortic segments were isolated from 6- and 14-week-old mice. Aortic segments were pre-contracted using phenylephrine (PHE; 10 µM), and their relaxation responses to acetylcholine (ACh; 1 nM- 10 µM) were assessed using wire myography with and without additional nicotine (100 μm). Relaxation (%) in the presence of indomethacin (10 µM, a COX inhibitor) (**A**). Relaxation in the presence of indomethacin and L-NAME (100 µM, NOS inhibitor). Maximal endothelial-dependent relaxation response (Emax, %) (**B**). Relaxation responses to sodium nitroprusside (SNP, 10 nM-10 µM) in the presence of indomethacin and L-NAME (**C**). All values are mean values ± SEM (*n* = 3–4 per group). *, *p* < 0.05; **, *p* < 0.01; ***, *p* < 0.001. Concentration-response curves (CRCs) were analyzed by Two-Way ANOVA and Bonferroni ‘s post-hoc test to compare control group in 6- vs. 14-week-old mice (1st row) and nicotine group 6- vs. 14-week-old mice (2nd row)

In the above-described experiments, among the constituent vape chemicals, nicotine was found to most consistently alter cellular responses. Further, vaped nicotine led to the most pronounced changes in aortic stiffness both in- and ex-vivo. While many published experiments have focused on specific cellular phenotype changes that could lead to structural vessel alterations (e.g. ECM architecture), aortic stiffening due to impaired endothelial response might also contribute to the observed in-vivo results. We examined aortic contractile responses, both endothelium-dependent and -independent relaxation. Murine descending thoracic aortic rings were harvested from 6- and 14-week-old male C57BL/6J mice and assessed by wire-myography with and without direct nicotine treatment. Contractile responses to phenylephrine did not significantly differ between nicotine treatment and control when comparing young vs. adult mice. Also, the endothelium-dependent relaxation responses to acetylcholine in the presence of indomethacin were not significantly altered (Fig. 5A). In the presence of both indomethacin and NOS inhibitor (L-NAME, 100 µM), relaxation responses were inhibited in both conditions and ages, showing that these relaxation responses were mediated by NO, but were not altered by nicotine (Fig. 5B). SMC sensitivity to NO and endothelium-independent relaxation were assessed using the exogenous NO-donor sodium nitroprusside (SNP). Again, no difference was found when comparing young vs. adult mice in the control group, yet aortic rings from young mice exposed to nicotine showed significantly reduced relaxation in the presence of L-NAME vs. aortic specimens from adult mice exposed to nicotine (Fig. 5C).

These results suggest that nicotine exposure in both age groups is associated with unchanged endothelium-dependent relaxation responses (mainly mediated by NO), but smooth muscle sensitivity to NO seems altered in young vs. aged mice in response to nicotine.

## Discussion

In the present study we demonstrate that short-term exposure to e-cig aerosol, especially when containing nicotine, increases markers of aortic stiffness in young (6 weeks) and adult mice (14 weeks), which is accompanied by reduced aortic elastin content, particularly in the infrarenal aorta. Further, we show that individual major chemical constituents of e-cig vapor affect cellular gene and protein expression in human vascular cells.

E-cigs have become commonly used devices among all age groups in the past decade, but especially among young adults [29]. In fact, e-cigs are now the most frequently used tobacco product among the young in the US [30]. However, in-vivo studies to date have rarely focused on impacts on young vasculature and have mostly evaluated mid- to long-term exposure. Also, few studies have compared differential responses to e-cig aerosol at different ages. Our results indicate an effect on aortic stiffness in young mice after only 3 weeks of exposure, especially when nicotine is added to the e-cig liquid.

While young vasculature is often described as having more reparative capacity, potentially attenuating harmful effects, we observed similar, and perhaps even more pronounced effects on aortic stiffening in young mice, along with a reduction in aortic elastin content. This may indicate the potential for serious long-lasting impacts on the vasculature, including predisposition for later-occurring pathological conditions like hypertension or atherosclerosis.

These findings are in line with a study by Alexander et al. who reported that long-term e-cig exposure (3–6 months) increases fibrosis in kidneys, heart and liver in mice, eventually leading to altered cardiovascular function with decreased heart rates and elevated blood pressure [22]. This is further backed by clinical studies reporting higher arterial stiffness in e-cig users, with acute increases directly after use [16, 31]. Taking our results in context, even short-term exposure may predispose to these later-onset effects.

Importantly, we especially observed increased aortic stiffness in PWV and myography measurements when nicotine was added to the e-liquid as demonstrated in Fig. 1A–D. This is in-line with a study investigating cardiac impairment in a model of e-cig exposure and hyperlipidemia, where ventricular function was impaired due to changes in cardiac structure including myofibrillar derangement, thinning, cardiomyocyte destruction, and mitochondrial hypertrophy, which only occurred when nicotine was present in the e-liquid [32]. A recent study exposing 8-week-old mice for 90 days to e-cig vapor observed comparable effects on vasculature compared to our study, with increased arterial medial thickness likely due to hyperplasia, and enhanced PWV [33]. Similar observations were made in our study, but with only a quarter of that exposure duration. We also observed a trend towards increased intimal thickness with nicotine aerosol exposure, though not significant (Fig. 1G).

In our experiments, e-cig aerosol exposure led to earlier increased PWV in adult mice compared to young mice, while pressure myography only showed significant effects on aortic stiffness in young mice. It should be noted that the largest confounding variable to pulse wave velocity measurement is mean arterial pressure (MAP) [34, 35]. Therefore, although we did not observe changes in MAP using the tail-cuff method, more precise techniques for assessing blood pressure could have potentially detected differences in MAP in response to vape exposure (Fig. 1E). This may have impacted the PWV results obtained and explain the divergent results.

We attribute these functional alterations primarily to remodeling of the extracellular matrix—specifically changes in elastin content. These modifications were evident in the thoracic aortas of both age groups, whereas in the infrarenal aorta, they were restricted to younger animals (Fig. 2A, B). While our in vitro model utilizes a simplified exposure protocol with concentrations guided by cytotoxicity data and established literature rather than physiological in vivo levels, the results provide mechanistic insight. Specifically, we observed a downregulation of ELN transcription alongside increased MMP2 expression (Fig. 3B), both of which were validated at the protein level (Fig. 3C) and via enzymatic activity assays (Fig. 4B).

Our results are supported by a study by Aboaziza et al. who described changes in extracellular matrix composition and aortic stiffness in offspring of e-cig exposed rats [36]. In that study, changes in aortic relaxation were linked to increased oxidative stress, which we also observed in ECs treated with e-cig chemical constituents (Fig. 4).

When we assessed the effects of different chemicals predominant in e-cig aerosol on endothelial cells, we observed an increase in gene expression of surface markers like ICAM, VCAM and IL-8, indicating endothelial activation (Fig. 3A, B). Notably, a study by Alakthar et al. utilizing a combination of e-cig exposure and AAV-induced hyperlipidemia found marked increase in soluble markers of endothelial cell activation (ICAM-1, E-selectin, P-selectin) in the serum of exposed mice [37]. Their 5-week-old mice were exposed using the same exposure system we employed (SCIREQ inExpose), with pod-mod vape (JUUL) for a total of 4 weeks; comparable experimental conditions to our study.

Notably our wire-myography experiments (Fig. 5) revealed unchanged endothelium-dependent relaxation responses (which are mainly mediated by NO), while the smooth muscle sensitivity to NO seemed to be altered in young vs. aged mice in response to nicotine in native aortic tissue. Both vascular ECs and SMCs express multiple α and β subunits of nicotinic acetylcholine receptors (nAChR) [38–40] making the vessel wall a direct target of nicotine. When Olfert et al. analyzed ex-vivo dose-response curves for phenylephrine, methacholine and sodium nitroprusside obtained from thoracic aorta ring segments following 8 months of exposure to e-cig aerosol they found the endothelium-mediated vasodilatory response to be impaired, while the non-endothelium-dependent response to sodium nitroprusside was not altered [20]. Our experimental set-up differed, since we exposed native aortic rings to nicotine, rather than examining aortic rings harvested from previously treated animals, therefore primarily examining short time-exposure-related effects. In this context, Singhrao et al. found vascular SMC cAMP production (and therefore murine aortic vasodilatory capacity) to be significantly reduced with nicotine exposure, which may be one explanation for our observed results [41]. Additionally, a clinical trial revealed that conventional smoking induces early impairment of endothelium-independent arterial dilatation in smokers compared to controls [42], while another clinical study found long-term e-cig users to have higher arterial stiffness but similar endothelial function compared with non-smokers [43].

In summary, our results indicate that even short-term exposure to e-cig aerosol leads to marked changes in aortic stiffness and elastin content, with young mice being affected to a similar, if not greater, extent compared to adults. Exposure to chemicals present in murine plasma and urine due to e-cig consumption led to changes in gene and protein expression in cultured ECs and VSMCs which may be in part responsible for the observed effects. Our results add more valuable information on the effects of e-cigs on the vasculature and suggest that they potentially may predispose to cardiovascular disease, especially when started at an early age.

## Limitations

Our study has several limitations. First, we were not able to identify underlying regulatory mechanisms leading to the observed changes in gene and protein expression which could potentially be responsible for the effects observed in the in- and ex-vivo functional assessment of aortic stiffness. This will need to be addressed in future studies, with focus on elucidating the mechanisms underlying ELN degradation and contribution of inflammatory cellular response in this context. Further, the in-vitro experiments were only conducted in significantly simplified experiments, and using only two commercially available cell lines, recognizing that large arterial vessels are composed of additional cell types which may display various interactions upon stimulation. A more complex in-vitro model might add valuable information in this regard. In addition, only male mice were used for the experiments in this study, potentially missing gender-attributed differential responses to e-cig exposure.

## Materials and Methods

### E-cig Aerosol Exposure in Mice

Animal protocols were approved by the Administrative Panel on Laboratory Animal Care at Stanford University (http://labanimals.stanford.edu/) or VA Institutional Animal Care and Use Committee and followed the National Institutes of Health and U.S. Department of Agriculture Guidelines for Care and Use of Animals in Research (protocol# TSA1819). All experiments were performed with C57BL/6J mice. Animals were purchased from The Jackson Laboratory (Bar Harbor, ME, USA). Male 6- and male 14-week-old C57BL/6J mice were exposed to e-cigarette aerosol containing 50% propylene glycol, 50% vegetable glycerol +/- 24 mg/ml liquid nicotine base for a total of three weeks with the SciReq InExpose system (SCIREQ, Montreal, Canada) in a whole-body chamber. Mice were exposed to a puff duration of 9 s per minute, for 60 min total on a daily basis. The device used was a third-generation e-cigarette (Shenzhen Joyetech Co., Shajing Town, China) with a temperature-controlled coil set to 230 °C. Control mice were exposed to room air only. Control animals were placed in the machine for the same daily duration without receiving any aerosol. Mice were monitored weekly for weight gain over the study period. After termination, heart weight and final body weight were also measured.

### Pulse Wave Velocity Assessment and Media Thickness

Mice underwent weekly examination for Pulse wave velocity (PWV) using ultrasound (Vevo 2100^®^ High-Resolution In Vivo Micro-Imaging System, VisualSonics) Here, global aortic stiffness was assessed by simultaneous tracking of the R-wave of the ECG and the pulse wave at two specific locations: the left subclavian artery (LSA) and the iliac bifurcation (bif). We determined the PWV as a ratio of the distance (d) and time (t) delay of the pulse wave between both locations. PWV was calculated as PWV = [d(bif)-d(LSA)]/[t(bif)-t(LSA)]. All measurements were conducted following the two-mean principle. The PWV measurements were obtained within one hour after E-cig exposure on the respective days.

Aortic media thickness was measured using the M-Mode ultrasound images acquired for PWV measurements for the infrarenal aorta, using the commonly available image analysis tool Fiji [44].

### Ex-Vivo Pressure Myography

Pressure myography was performed as previously described [45]. In short, the thoracic and abdominal (infrarenal) aorta were explanted from vapor-exposed mice at the end of exposure. The midparts of the descending thoracic and infrarenal aorta were dissected and further processed. Specimens of approximately 0.6–0.8 cm in length were placed on specially designed stainless-steel cannulas and secured with silk surgical suture (10 − 0). Aortic segments were mounted in the heated vessel chamber of a pressure arteriograph system (Model 100P, Danish Myotechnology, Copenhagen, Denmark) and extended to in vivo length. Physiological saline solution at 37 °C was used to fill the vessel chamber and for aortic perfusion. Subsequently, aortic segments were pressurized from 0 to 144 mmHg in 18 mmHg increments, and the vessel’s outer diameter was simultaneously tracked by continuous computer video analysis. The strain (S) was calculated as a ratio of outer diameter at baseline (D_b_) to outer diameter at every given pressure level (D_p_) (S = (D_p_−D_b_)/D_b_).

### Blood Pressure Measurement

Mice in both age groups were monitored for changes in blood pressure during the study period. One week prior to the start of the measurements, mice were placed in the tubes once per day to allow acclimatization. Measurements were performed after daily puff exposure over the 3-week time course of the experiment using a non-invasive cuff-tail system (CODA noninvasive system, Kent Scientific Corporation, Torrington, USA).

### Urine/Plasma Analysis

A subset of mice of 14-weeks-of-age was exposed to e-cig aerosol containing 50% propylene glycol, 50% vegetable glycerol with 24 mg/ml nicotine and sacrificed within 30 min after exposure, to collect blood and urine. Blood was collected from each individual to EDTA tubes by cardiac puncture and further processed for plasma isolation. Urine samples were taken by bladder puncture and pooled for analysis, due to the small sample amount per animal. Samples were stored at −80 °C and subjected to high-pressure liquid chromatography with tandem mass spectrometry quantification of major chemicals contained in vapor, namely nicotine, acrolein, formaldehyde, acetaldehyde and propylene-glycol performed by Climax Laboratories inc. (San Jose, CA, USA).

### Histology

A subset of mice underwent the e-cig-vapor exposure protocol described above and was sacrificed after the exposure period. The aorta was flushed with cold saline through heart-puncture and thoracic and infrarenal segments transferred to 4% paraformaldehyde (24 h), cryoprotected in 25% sucrose in phosphate-buffered saline (PBS) (24 h), 50% sucrose in PBS (24 h), and embedded in optimal cutting temperature (OCT) compound (Tissue-Tek). Cryosections of 7 μm thickness were systematically collected and afterwards stained for HE and Elastica Van Gieson using a standardized protocol. Sectioning and staining of the embedded specimens were performed by Histotek (Fremont, CA, USA).

### Elastin Quantification

Images from aortic cross-sections subjected to Elastica Van Gieson staining were analyzed to quantify elastin content using Fiji, as shown in Fig. 2A. Briefly, the relative amount of elastin fibers in the two-dimensional histological image was defined. The ratio between the area of the elastin fibers and the total area of the section was used. The “Color Deconvolution” plug-in was used to automatically display the area in pixels. The different structures were separated based on their different red-green-blue (RGB) values, which were represented by the coloring. The selection of two to three colors made it possible to display the RGB values. The values were integrated into the source code of the plug-in and the images were separated based on the newly determined vector. The captured areas were ultimately displayed in white and the background in black. The number of pixels in the white areas, i.e. the size of the areas to be measured, was determined using the measurement function. The results obtained were used to calculate the ratio between elastin fibers and the total cut surface in order to estimate the relative amount of elastin.

### Cell Culture

Human aortic smooth muscle cells (haoSMC, Cell Applications, Inc. San Diego, USA) were propagated in growth media [DMEM with 20% fetal bovine serum (FBS) and 1% Pen/Strep per standard protocols (passage #4–6). Human aortic endothelial cells (haoECs, PromoCell, Heidelberg, Germany) were cultivated in Endothelial Cell Growth Medium (PromoCell, Heidelberg, Germany) per standard protocol (passage #3–5). Cells were cultivated at 37 °C and 5% CO2 (HERAcell240, Heraeus, Hanau, Germany). At 90% confluence, the cells were sub-cultured using 0.05% trypsin/0.02% ethylenediaminetetraacetic acid (EDTA) (PAN Biotech GmbH, Aidenbach, Germany). Morphological cell assessment was performed using phase-contrast microscopy (Olympus CKX41, Olympus, Shinjuku, Japan).

#### Cell Viability

1 × 10^5^ haoECs or 1 × 10^4^ haoSMCs were seeded into a 96-well plate (Sarstedt, Germany) and 100 µL media was added. The cells were incubated overnight at 37 °C and 5% CO2 inside an incubator (HERAcell240, Heraeus, Hanau, Germany). The next day, cells were exposed to the respective treatments for 24 h. Thereafter, 10 µL of WST reagent was added to each well. After 2 h incubation in dark, absorbance was measured photometrically using a VICTOR™ X4 Multilabel Plate Reader (PerkinElmer, Baesweiler, Germany) at 450 nm.

### RT-PCR and Western Blotting

To study transcriptional and protein expression changes, haoECs and haoSMCs were exposed to the respective treatments for 24 h. Total RNA isolation from cell cultures was performed using the RNeasy Plus Kit (Qiagen, Hilden, Germany) following the manufacturer’s instructions. RNA was eluted in 50 µL RNase-free water (Qiagen, Hilden, Germany). The RNA concentration was determined spectrophotometrically using a Nanodrop (NANODROP 2000c Spectrophotometer, Thermo Scientific™, Waltham, MA, USA) at 260 and 280 nm. Complementary DNA (cDNA) synthesis was performed using high-capacity cDNA Reverse Transcription Kit (Thermo Fisher Scientific, Waltham, MA, USA) following manufacturer’s instructions, with 500 ng RNA input. The cDNA protocol consisted of the following: annealing at 25 °C for 10 min, extension at 37 °C for 120 min, and inactivation of reverse transcriptase at 85 °C for 5 min using a state-of-the-art thermocycler. For RT-PCR experiments, the cDNA input was 1 µL. TaqMan qRT-PCR assay was used to quantify the mRNA levels. Specific oligonucleotide primers for Collagen3A1 [COL3A1, # HP200076], Matrix-metalloproteinase 7 [MMP2 # HP207826], Tissue Inhibitor of Metalloproteinases 2 [TIMP2, # HP206805], Transforming Growth Factor Beta Receptor 2 [TGFBR2, # HP206790], Elastin [ELN, # VHPS-2951], Intercellular adhesion molecule 1 [ICAM1, # HP200186], Vascular cell adhesion molecule 1 [VCAM1, # HP230503], Cluster of differentiation 80 [CD80, # HP208372], Interleukin 8 [IL8, # HP200551], Interleukin 6 [IL6, # HP230503] were obtained from ORiGEN (ORiGEN, Austin, TX, USA) or BioMol (BioMol. Hamburg, Germany). The data were normalized to GAPDH (# HP205798) and fold changes were calculated using the ΔΔCt method, as previously described [46].

For Western blotting, treated cells were lysed in RIPA buffer. In detail, protein of each sample was separated by SDS-PAGE (12% acrylamide) and transferred to 0.2 μm nitrocellulose membranes (Fisher Scientific, Waltham, MA, USA) and blocked in 1% BSA/1% nonfat dry milk for 1 h at room temperature. ELN and COL3A1 antibodies (ELN: rabbit polyclonal, Bioss, Woburn, MA, USA, catalog# bs-1756R, 1:1000 dilution; COL3A1: rabbit polyclonal, Thermo Scientific, Waltham, MA, USA, catalog# PA5-34787, dilution 1:2000) were used and the samples were incubated at 4 °C overnight. The next day, the membranes were washed and incubated with secondary peroxidase-conjugated antibodies at a 1:20.000 dilution (Fisher Scientific, Waltham, MA, USA) for 1 h at room temperature. Chemiluminescence was detected with the Clarity Max Western ECL substrate (Bio-Rad, Hercules, CA USA) using ChemiDoc (Bio-Rad Hercules, CA, USA) and normalized to ß-Aktin Alexa 647 (ß-Actin: rabbit monoclonal, Cell Signaling Danvers, MA, USA catalog# 8584, dilution 1:3000).

### ROS Assay

Treatment-induced oxidative stress and superoxide levels were quantified using the ROS/Superoxide Detection Assay Kit (Abcam/ab139476). Oxidative stress was detected as fluorescein (excitation/emission = 490/525 nm) and superoxide as rhodamine (excitation/emission = 550/620 nm). HaeEC at passage 10 were seeded into a fluorometry-compatible 96-well plate (1 × 10⁴ cells in 100 µl medium per well) 24 h before treatment. Each condition was tested in eight replicates. Chemical exposure was initiated 24 h prior to detection to ensure consistent analysis. Detection followed the manufacturer’s protocol. Pyocyanin (400 µM) and N-acetyl-L-cysteine (5 mM) served as positive and negative controls, respectively. According to the protocol, incubation with the ROS/Superoxide Detection Mix after ROS induction should last 30–60 min. To improve data reliability, measurements were taken at 30, 45, and 60 min using a multimode reader at both wavelengths.

### MMP Activity

HaoSMCs (passage 3) were seeded in a 96-well fluorometric plate at 1 × 10^4 cells per well in 100 µl culture medium. Each condition was performed in six replicates. After 24 h, cells were treated with five different chemicals for another 24 h, using concentrations described previously. MMP activity was measured following the manufacturer’s protocol (ab112146). Culture medium was removed and replaced with 25 µl of assay buffer. For buffer controls, six wells without cells received 25 µl buffer. Then, 25 µl of 2x APMA working solution was added to each well (final volume: 50 µl) and incubated at 37 °C for 1 h to activate MMP-2. Additional control wells received 50 µl assay buffer without APMA. After incubation, 50 µl of diluted MMP Green Substrate solution was added per well and incubated at room temperature for 45 min. Fluorescence was measured at 485 nm (excitation) and 525 nm (emission).

### Ex-Vivo Wire Myography

For wire myograph experiments, C57BL/6J wild type mice from Janvier Labs (Saint-Berthevin Cedex, France), aged 6-weeks and 14-weeks, were euthanized via cervical dislocation. Procedures were approved by local committee at the central animal facility (ZETT) at Heinrich-Heine-University Düsseldorf, protocol #O63/18.

The rib cage was opened and the thoracic aorta was carefully dissected from the periaortal adipose tissue without stretching or compression. The thoracic aorta was then cut into 2 mm long aortic rings, which were incubated in 100 nM nicotine or Krebs-Ringer bicarbonate buffer (KB) for one hour. KB contained 115 mM NaCl, 4.7 mM KCL, 1.4 mM MgSO4, 5 mM NaHCO3, 1.2 mM KH2PO4, 1.1 Na2HPO4, 1.0 mM CaCl2 and 5 mM glucose. The KB solution was continuously aerated with 95% O2 and 5% CO2 and maintained at 37 °C. After incubation the aortic rings were carefully mounted onto a wire-myograph system (Automated Multi-Myograph System 630 MA, Danish Myotechnology, Denmark). The aortic rings were distended stepwise to 9.8 mN and incubated for 45 min in KB. Before initiation of contraction and relaxation responses the aortic rings were incubated with 10 µM indomethacin (INDO, Sigma Aldrich), which was dissolved in ethanol and NG-nitro-arginine methyl ester (L-NAME, Sigma Aldrich), dissolved in KB. For the dose-response curves phenylephrine (PHE, Sigma Aldrich) and acetylcholine (ACH, Sigma Aldrich) were dissolved in KB and added to the chambers in ascending concentration from 10 − 9 µM to 10 − 5 µM. Sodium nitroprusside (SNP, Sigma Aldrich) was also dissolved in KB and added stepwise from 1010 µM to 10 − 5 µM.

### Statistics

Data are presented as mean ± SEM. Groups were compared using Student’s t-test (two-tailed) for parametric data. When comparing multiple groups, data were analyzed by ANOVA with Bonferroni’s post-test. Sequential measurements were analyzed by One-Way Repeated Measures ANOVA. Paired t-testing was performed utilizing Wilcoxon matched-pairs signed rank test with Spearman effectiveness testing. All statistic testing and graph composition was done using GraphPad Prism software (San Diego, USA, Version 10.0.1). A value of *P* ≤ 0.05 was considered statistically significant.

## Supplementary Information

Below is the link to the electronic supplementary material.

Supplementary material 1 (DOCX 1287.3 kb)

## Data Availability

All data supporting the findings of this study are available within the paper and its Supplementary Information. Additional primary data can be made available by the authors upon reasonable request.
