# Supplementary material for: E-cigarette Aerosol Containing Nicotine Increases Aortic Stiffness in Young Mice
Source: Cardiovasc Toxicol. 2026 Jul 8;26(7):72. doi: 10.1007/s12012-026-10156-1 (PMC13342398; doi:10.1007/s12012-026-10156-1)
Supplement: Supplementary file 1 — Supplementary material 1 (DOCX 1287.3 kb) [file 12012_2026_10156_MOESM1_ESM.docx]

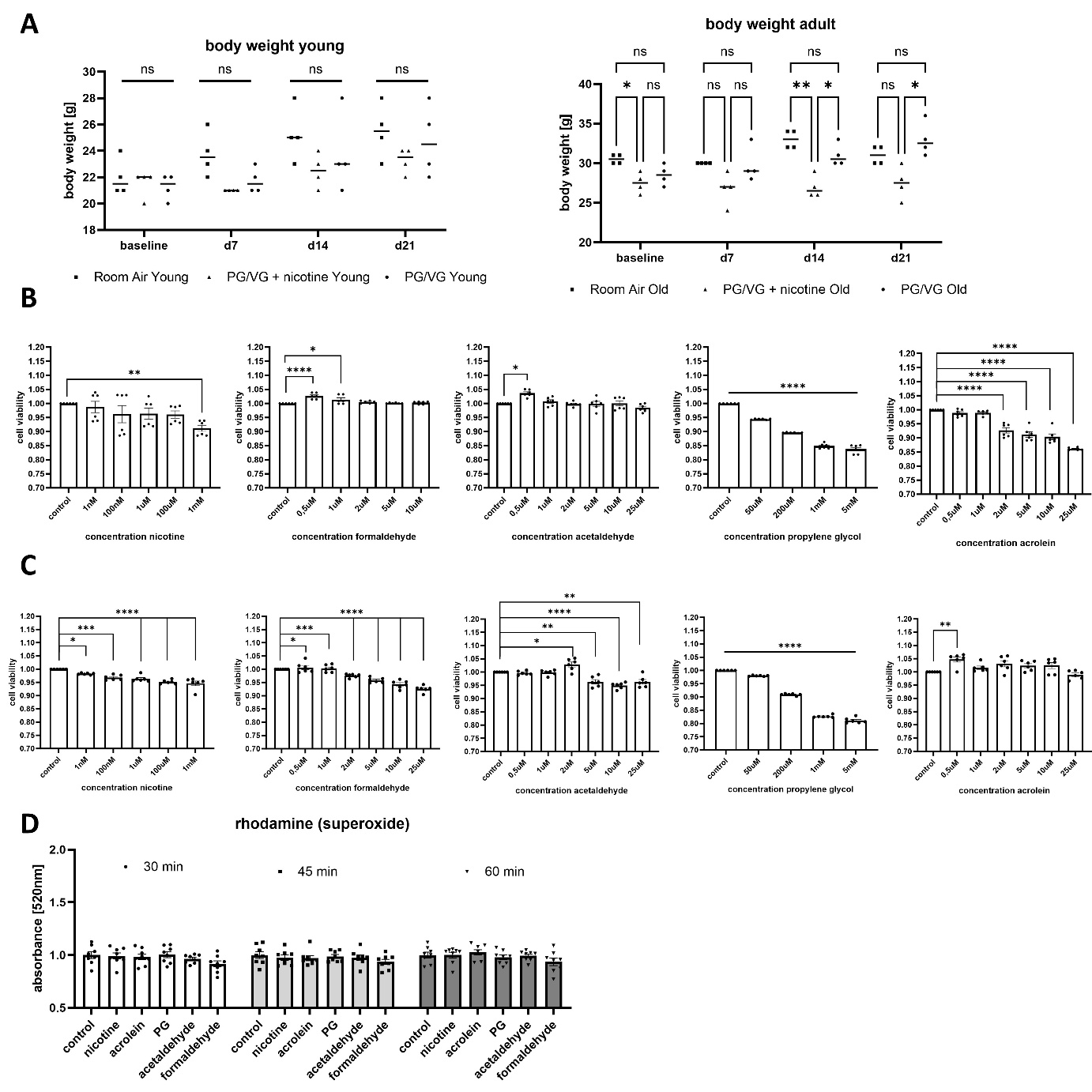


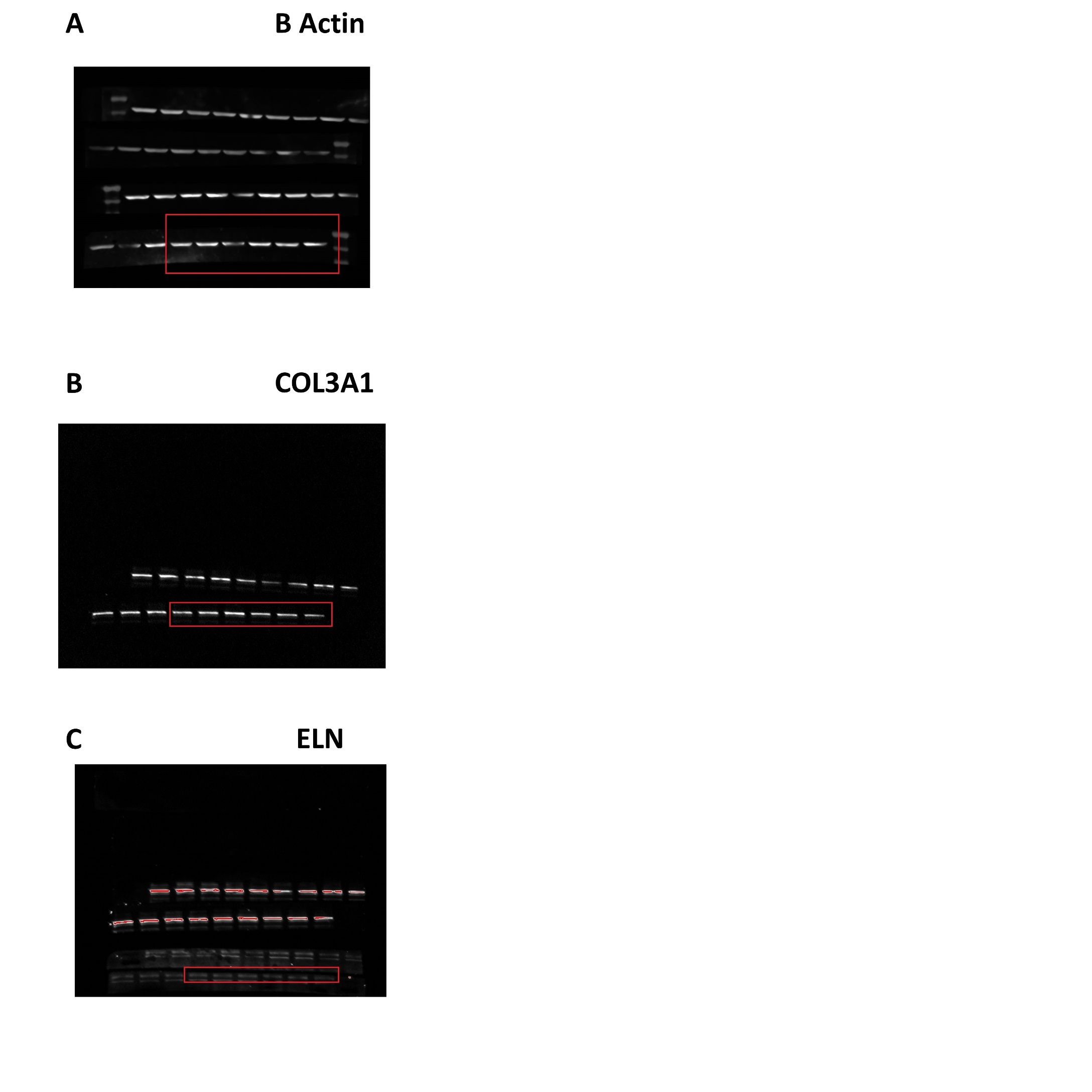


**Suppl. Figure 1. Body weight over time, Effects of e-cig aerosol constituting chemicals on cell viability in haeEC and haoSMC. Superoxide generation in haoECs not affected.** Body weight in mice in different treatment groups over the 3-week period in 6 week-old (left) and 14-week-old (right) mice. n=4 per group (**A**). Cell viability assessed by WST-assay in human endothelial cells after treatment with e-cig aerosol constituents in increasing concentrations (**B**), n=6 per group. Cell viability assessed by WST-assay in human aortic smooth muscle cells after treatment with e-cig aerosol constituents in different concentrations (**C**) n=6 per group. Following 24-hour exposure to several e-cig aerosol constituting chemicals, human aortic ECs were assessed for superoxide production by fluoresce absorbance measurements (**C**) n=8 per group. Data shown as mean with SEM, *=p<0.05; **=p<0.01; ***=p<0.001; ****=p<0.00001 vs. untreated control assessed by ANOVA with multiple comparison.

**Suppl. Figure 2. Uncropped gels of Western Blot gels shown in Figure 3 E, F.** ELN and COL3A1 protein content were assessed using Western blotting in human aortic SMC’s previously treated with chemicals as described in the materials and methods section. Red rectangle shows part of gel displayed in Figure 3 C and D of main manuscript; Β-Actin (45kD**, A**) for COL3A1 (180kD, **B**) and ELN (80kD, **C**).
